# Behavioural activation interventions for depressed individuals with a chronic physical illness: a systematic review protocol

**DOI:** 10.1186/2046-4053-2-105

**Published:** 2013-11-16

**Authors:** Sarah Harris, Paul Farrand, Chris Dickens

**Affiliations:** 1 Mood Disorders Centre, Psychology, College of Life and Environmental Sciences, University of Exeter, Exeter EX4 4QG, England; 2 University of Exeter Medical School, College House, St Luke’s Campus, Exeter EX1 2 LU, UK

**Keywords:** Behavioural activation, Depression, Chronic physical illness, Systematic review

## Abstract

**Background:**

Depression is common in people with chronic physical illness and is associated with worse medical outcomes. Cognitive behavioural therapy and problem-solving improve depression, although usually have small to moderate effects among people with chronic physical illness. Behavioural activation interventions for depression, which aim to increase positive reinforcement from the environment by encouraging individuals to increase pleasant/rewarding activities, have been reported to be equivalent to cognitive behavioural therapy. However, the effectiveness of behavioural activation interventions for depression in individuals with chronic physical illness is unclear. The aims of this systematic review are to identify the extent to which different forms of behavioural activation have been used as a treatment for depression in this population, examine the effectiveness of the interventions, and identify any adaptations which have been made specifically to the interventions for individuals with a range of chronic physical illnesses.

**Methods/Design:**

Electronic databases will be systematically searched using terms relevant to behavioural activation and depression, and the subset of studies in people with chronic physical illnesses will be identified by manual searching. References and citations of eligible studies will be searched and experts in this field will be contacted to identify additional papers. All study designs will be included in this review to allow for a more extensive identification of the extent of different forms of behavioural activation interventions. The different forms of behavioural activation and the specific chronic physical health conditions for which this intervention has been used will be reviewed narratively. For the effectiveness of the interventions, if sufficient randomised controlled trials have been undertaken the results will be meta-analysed. Non-randomised studies will be narratively synthesised and adaptations to the interventions will also be narratively reviewed.

**Discussion:**

The findings will inform the design, development and subsequent evaluation of a behavioural activation intervention for depression in people with a chronic physical illness. PROSPERO registration number: CRD42013004500.

## Background

Depression affects between 7.9% and 23.0% of people with chronic physical illnesses [1,2] and is associated with worse medical outcomes, such as more physical symptoms [3], greater healthcare use, increased work disability [4] and higher mortality [5,6]. Therefore, treating depression in individuals with chronic physical illnesses, with interventions which are effective, is important for improving health-related quality of life in this population. Treating depression as a co-morbidity in patients with chronic illness has increasingly become a priority with respect to mental health provision [7]. Conventional psychological treatments for depression, such as cognitive behavioural therapy (CBT) and problem-solving treatment, can improve depression, although effects in individuals with chronic physical illnesses have generally been small to moderate [8-10]. For some therapies no demonstrable benefits of the psychotherapeutic intervention are found [11]. One psychosocial nursing intervention was far from clinically significant and was associated with worsening of psychological state by increasing distress in some patients [12]. Consequently, there is scope to develop and evaluate novel psychological treatments for depression that are appropriate for use in people with chronic physical illness.

Behavioural activation (BA) for depression has received interest and empirical support after a component analysis found it was equally as efficacious as CBT [13]. The basic conceptual foundation for BA is based upon the original behavioural models of depression [14,15], which propose depression to be a result of a lack of response-contingent positive reinforcement [14] and emphasise the importance of a functional analysis of behaviour and the role of avoidance and escape behaviours in depression [15]. The main aim of BA treatment approaches is to help patients with depression overcome sources of negative reinforcement that maintain their inactivity and increase access to sources of positive reinforcement from the environment through pleasant/rewarding activities. There are, however, variants in the BA approach [16] with slightly varied treatment protocols with differing strategies of change and lengths of treatment [17].

Pleasant activities interventions (for example, see [18]) involve monitoring and scheduling pleasant activities, with some protocols also including social skills training. A meta-analysis found a large overall effect size for activity scheduling, and in comparisons, activity scheduling and cognitive therapy (CT) were reported to be equally effective, at post-test and follow-up [19]. Self-control interventions (for example, see [20]) include monitoring activities and mood, setting of goals, performance self-evaluation and self-administering rewards [16]. An emphasis upon self-management skills helps individuals to progress towards goals that are personally important and engage more in reinforcing behaviours.

Contextual BA interventions (for example, see [21,22]) use activity scheduling, self-monitoring, graded task assignment and role-playing. The function of behaviour is very important in contextual BA interventions, utilising TRAP (Trigger, Response, Avoidance Pattern) and TRAC (Trigger, Response, Alternative Coping) as the basis of a functional analysis to identify the function of avoidance behaviours and help the selection of alternative behaviours to improve depression. Once appropriate activities are identified, clients are encouraged to assess the function and how different types of activities serve them, choose to either avoid or activate, engage in the chosen activity, integrate new activities into their lifestyle, observe the outcome upon their mood and never give up (ACTION) [22]. Focused activation is also important in contextual BA, not using a broad class of behaviours which are assumed to be positively reinforcing, but working with the individual to understand current activities, and which activities to engage in [21]. For more severely depressed individuals contextual BA is comparable in efficacy to antidepressant medications and more efficacious than CT [23].

Behavioral activation treatment for depression (BATD) [24,25] consists of assessing the function of depressed behaviour, weakening access to positive and negative reinforcement for depressed behaviour, establishing patient rapport and a systematic activation approach involving self-monitoring, identifying behavioural goals and an activity hierarchy [17]. Unlike the contextual approach, this intervention does not focus significantly on helping individuals with a functional analytic interpretation of their behaviours - this is secondary to the activation component [17]. BATD is reported to be a clinically significant treatment in an inpatient psychiatric hospital [26] and produces significantly greater reductions in depression in university students compared to no treatment [27].

Meta-analyses have shown BA interventions as superior to brief psychotherapy and supportive counselling [28] and equivalent to CT and CBT [16,19,28]. Apart from CT/CBT, BA interventions may be more successful for maintaining increases in wellbeing at follow-up periods of up to 3 months than other psychological interventions [29] and for depression is equivalent to CT/CBT for up to 24 months [16]. BA interventions are time-efficient, relatively uncomplicated [17,26], easy to implement [24] and cost-effective. Considering the cost utility, there is a 97% probability that BA delivered by non-specialists to adults with depression from general practice or primary care mental health services, is more cost-effective than usual care at a threshold value of £20,000/quality-adjusted life-year [30].

However, few studies have investigated the effects of BA interventions in depressed people with chronic physical illness, and there are currently no systematic reviews for this intervention in this population. With the focus of the treatment based on resuming rewarding activities and not on changing cognitions, BA interventions may be acceptable to people with chronic physical illnesses. This systematic review therefore seeks to identify the extent to which BA interventions have been used as a treatment for depression in individuals with a chronic physical illness, examine the effectiveness of these interventions and identify whether any adaptations have been made to BA specifically to accommodate the needs of people with chronic physical illnesses. Our findings will then inform the design, development and subsequent evaluation of a BA intervention for depression in people with a chronic physical illness.

### Aims

The aims of this systematic review are to: (1) identify the extent to which different forms of BA have been used as a treatment for depression in adult patients with a range of chronic physical illnesses; (2) examine the effectiveness that different forms of BA have across a range of chronic illnesses by conducting a meta-analysis for chronic physical illnesses where enough studies have been undertaken, or by narrative synthesis if sufficient studies are not found; and (3) undertake a narrative synthesis to identify any adaptations that have been made to any BA intervention when used to treat depression in patients across a range of chronic physical illnesses.

## Methods

This systematic review will be conducted following the Centre for Reviews and Dissemination (CRD)’s guidance for undertaking reviews in health care [31] and the Economic and Social Research Council (ESRC) Research Methods Programme’s guidance on narrative synthesis [32]. The review will be reported following the Preferred Reporting Items for Systematic Reviews and Meta-Analysis (PRISMA) statement [33]. This review is registered with the PROSPERO International Prospective Register of Systematic Reviews, registration number: CRD42013004500.

### Inclusion and exclusion criteria

#### Population

Studies will be eligible for inclusion in this review if they include adults (aged ≥16 years) with: (1) a diagnosis of depression/a depressive disorder (according to a diagnostic interview) or an elevated level of depressive symptomatology (scoring above a cutoff on a validated self-report scale); and (2) a chronic physical health condition. A chronic physical health condition is identified as a long term condition that cannot be cured, but is controlled by medication and/or other therapies/treatment (see [34]).

#### Intervention

To be considered a BA intervention, the intervention should be based upon a behavioural model of depression, with the main aim of the intervention being to increase positive reinforcement from the environment using strategies to encourage individuals to increase pleasant/rewarding activities, and/or reduce escape and avoidance behaviours by increasing a range of activities. The intervention should not target cognitions as a main mechanism of change.

#### Comparators

As all study designs are included in this review, a range of comparators will be included to form the selection criteria. For experimental studies, control groups in controlled studies may receive treatment as usual or other psychological or pharmacological interventions. For quasi-experimental, observational or qualitative studies no comparator may have been adopted.

#### Outcomes

The outcomes of interest for aim (2) are scores relating to whether the patient is still diagnosed with depression following a diagnostic interview or scores on a validated self-report scale examining severity of depressive symptomatology. With respect to aim (3) there are no specific outcomes of interest, instead analysis is based upon a qualitative synthesis regarding any adaptations made to the BA protocol employed to make it suitable to treat depression in patients with chronic physical illnesses.

#### Study design

Due to an expected small number of randomised controlled trials (RCTs) and given the focus of the systematic review upon either a meta-analysis or narrative synthesis, there is no exclusion based upon methodology employed in the studies. As such all study designs will be included in the search and selection including: experimental studies (RCTs, quasi-randomised trials, controlled clinical trials) quasi-experimental studies (interrupted time series, before-and-after studies), observational studies (cohort studies, case–control studies, case series) and qualitative studies.

#### Other limiters

Studies which are eligible for inclusion must have an English translation available publically. The Cochrane Library will be searched from inception, Medline from 1946, Cumulative Index to Nursing and Allied Health Literature Plus (CINAHL Plus) and The Allied and Complementary Medicine Database (AMED) from 1950, Excerpta Medica DataBase (EMBASE) from 1974 and PsycINFO from 1967. If applicable to databases, a human filter will also be applied.

### Search strategy

The following electronic databases will be searched with the predefined search strategy attached (Additional file 1): PsycINFO (Ovid); Medline (Ovid); EMBASE (Ovid SP); The Cochrane Library; CINAHL Plus (EBSCO); and AMED (EBSCO). Alterations to the MeSH terms included in the search strategy will be undertaken to ensure consistency with the database being searched. Additional published and unpublished research will be identified by searching the citations and references, and by contacting the authors of eligible papers. To avoid publication bias all relevant studies will be assessed, including published articles, conference abstracts, book chapters and any unpublished research supplied by contacted authors.

The search strategy will include ‘Behavioural Activation’ and ‘Depression’ , and terms associated with these (see Additional file 1). Preliminary work (SH) has however identified that there are no acceptable search terms or definitive list of sufficient sensitivity and specificity to identify studies of subjects with all chronic physical illnesses. Consequently, to avoid missing relevant research, terms associated with chronic physical illnesses will not be included in the search strategy and the subgroup of studies of subjects with chronic physical illnesses will be identified by manual searching of the titles identified through the search.

### Study selection

The initial electronic search will be conducted by one researcher (SH). The electronic searches for different databases will be combined using EndNote X6 and duplicates will be identified and deleted (SH). The titles and abstracts of studies identified through electronic searching will be screened to identify studies that are potentially eligible for inclusion. Screening will aim to restrict the papers to be inspected in full text, but will ensure all papers that have data which are potentially relevant are retained to the full text stage.

The same reviewer will manually screen the titles for chronic physical illnesses and the abstracts of all studies included after title screening will then be screened by two reviewers. Any discrepancies will be discussed to ensure the sensitivity of screening. All full text versions of papers that remain potentially relevant will be screened by two reviewers independently. This will identify studies that are definitely eligible for inclusion. The findings of the two researchers will be compared. Discrepancies in studies identified as relevant during this process will be resolved by discussion and the involvement of a third reviewer (CD/PF) where consensus cannot be reached. The number of studies at each stage and the reasons for any exclusion of papers at the full text stage will be recorded in a PRISMA flow diagram [33].

### Quality assessment

The quality of all eligible studies will be assessed by the main reviewer (SH) and checked by a second reviewer. RCTs will be assessed using the Cochrane Collaboration’s Risk of Bias tool [35]. Non-randomised studies will be assessed using the Downs and Black checklist [36] for measuring study quality. Qualitative studies will be assessed using the Critical Appraisal Skills Programme (CASP) checklist for qualitative studies [37]. Any discrepancies will be discussed and a third reviewer (CD/PF) will be involved if necessary.

### Data extraction

All data will be extracted by two researchers using standardised electronic data extraction forms (Additional file 2, adapted from [38]), which were developed for this systematic review and based on guidance from the CRD [31]. Data extracted will include characteristics of the study methodology, participants, statistical approaches and results. Details of the intervention components will also be extracted, including: BA components, mode of delivery, who the intervention is delivered by, training received by the practitioner delivering the intervention, whether the intervention is individual or group sessions, group size for group-based interventions, duration of intervention, number of sessions, length of sessions, treatment setting, whether the treatment is manualised (protocol driven) and measurement of treatment integrity. The extraction of intervention components will be used to identify any adaptations to the interventions. The data extraction form will be piloted on a sample of the studies that have been included after full text screening, and modified accordingly [31]. Findings from data extractions from the two researchers will be compared and discrepancies resolved by discussion and involvement of a further reviewer (CD/PF) where consensus cannot be reached. The data extraction forms will allow sufficient details to be extracted so the ‘Risk of Bias’ tool can be completed.

### Data synthesis and analysis

Aim (1) will be addressed with a narrative synthesis involving tables and text of the different forms of BA for depression in individuals with a range of chronic physical health conditions. As all chronic physical illnesses will be included in this review, the specific physical health conditions of patients in the studies will also be recorded.

For aim (2) the effectiveness of the BA interventions will be analysed dependent upon study design. If sufficient RCTs have been undertaken, findings comparing the effects of BA versus the appropriate comparator on depression will be meta-analysed [39] using Comprehensive Meta-Analysis Version 2.0 [40]. Effect sizes will be calculated for each independent trial using standardised mean differences or odds ratios, depending on the predominant way results are presented in included trials. Only one effect per independent study will be included in the meta-analysis. In situations where results for a single population are presented in multiple publications, a single effect size only will be calculated. Where studies present effects on depression at multiple follow-up times, a single follow-up period will be chosen for inclusion in the meta-analysis, most probably that closest to the median of the other studies to reduce heterogeneity. Effect sizes will be pooled using random effects models, weighted using the inverse of the variance [31]. Results will be presented in forest plots with the combined effect (95% confidence intervals). Heterogeneity among included studies will be investigated using Cochran’s Q test and the I^2^ statistic [41,42]. If sufficient data are available, variation in effect size across the characteristics of the study population and the intervention will be calculated using random effects, univariate meta-regression (continuous variables), and the analog to Analysis of Variance (categorical variables). Publication bias will be assessed using funnel plots and Eggers’ regression method [43], but will only be conducted if 10 or more trials are included [44]. Sensitivity analyses will be conducted to investigate the influence of study quality on the findings of the meta-analysis. Consistent with other meta-analyses and meta-regressions [10,45] the primary quality measure will be a binary measure of allocation concealment [46].

If there is not a sufficient number of RCTs and for any other non-randomised study designs, the effectiveness of BA interventions will be narratively reviewed following the guidance on the conduct of narrative synthesis [32]. Non-randomised studies will be reviewed this way and not meta-analysed due to expected greater heterogeneity due to the potential for methodological diversity and risk of biases through poor design and execution of the studies [47].

For aim (3) any adaptations that have been made to the BA interventions for individuals with chronic physical illnesses will be reviewed narratively. In the event that there are a suitable number of studies the synthesis will be undertaken by each chronic physical illness independently. The inclusion of all study designs, including qualitative research, will be undertaken to facilitate more detailed descriptions of any adaptations made.

## Discussion

Currently there are no systematic reviews that have examined the use of different types of BA when used to treat depression in individuals with chronic physical illness. Based upon the Medical Research Council’s (MRC) guidance [48], this systematic review will clarify and further develop theoretical underpinnings for the use of BA interventions in depressed individuals with chronic physical illnesses and identify adaptations that have been made to BA interventions specifically for this population. The findings from this systematic review will inform the development and subsequent piloting of a BA intervention for depressed individuals with a chronic physical illness.

## Abbreviations

AMED: The Allied and Complementary Medicine Database; BA: Behavioural activation; BATD: Behavioral activation for depression; CASP: Critical appraisal skills programme; CBT: Cognitive behavioural therapy; CINAHL: Cumulative Index to Nursing and Allied Health Literature; CRD: Centre for Reviews and Dissemination; EMBASE: Excerpta Medica database; ESRC: Economic and Social Research Council; MRC: Medical Research Council; PRISMA: Preferred reporting items for systematic reviews and meta-analysis; PROSPERO: International Prospective Register of Systematic Reviews; RCT: Randomised control trial; TRAC: Trigger, response, alternative coping; TRAP: Trigger, response, avoidance pattern.

## Competing interests

The authors declare they have no competing interests.

## Authors’ contributions

SH designed the study protocol and wrote the manuscript. PF and CD also contributed to the design of the protocol and were substantially involved in improving the drafts of the manuscript. All authors read and approved the final manuscript.

## Supplementary Material

Additional file 1 Ovid SP Embase search strategy. Click here for file

Additional file 2 Data extraction form to be used on Microsoft Excel. Click here for file
