# Supplementary material for: Behavioural activation interventions for depressed individuals with a chronic physical illness: a systematic review protocol
Source: Syst Rev. 2013 Nov 16;2:105. doi: 10.1186/2046-4053-2-105 (PMC3843584; doi:10.1186/2046-4053-2-105)
Supplement: Additional file 1 — Ovid SP Embase search strategy. [file 2046-4053-2-105-S1.pdf]

Ovid SP Embase search strategy

Ovid SP Embase from 1974 to 2013 January 10

Search conducted 14th January 2013

Limit to Human

1) (behavio$ adj activati$).ti,ab. (490)
2) (activity adj scheduling).ti,ab. (27)
3) (pleasant event$ or pleasant activit$ or daily diar$).ti,ab. (1,645)
4) (behavio$ adj therap$).ti,ab. (13,897)
5) Exp behavior therapy/ (33,404)
6) (behavio$ adj intervention$).ti,ab. (5,638)
7) 1 or 2 or 3 or 4 or 5 or 6 (45,408)
8) (depression or depressive or depressed or depressive disorder or mood disorder).ti,ab. (238,371)
9) Exp Depression/ (249,639)
10) Exp depressive disorder/ (249,639)
11) 8 or 9 or 10 (332,053)
12) 7 and 11 (9,697)
