# Supplementary material for: Behavioural activation interventions for depressed individuals with a chronic physical illness: a systematic review protocol
Source: Syst Rev. 2013 Nov 16;2:105. doi: 10.1186/2046-4053-2-105 (PMC3843584; doi:10.1186/2046-4053-2-105)
Supplement: Additional file 2 — Data extraction form to be used on Microsoft Excel. [file 2046-4053-2-105-S2.pdf]

Data Extraction Form to be used on Microsoft Excel

Researcher performing extraction:
Date of data extraction:

Study Identification Features	
Unique Study Identifier	
Title	
Authors	
Year of publication	
Citation	
Publication type	
Country of origin	
Source of funding	

Study Characteristics & Quality		
Aims/objectives		
Study design		
Study inclusion/exclusion criteria		
Recruitment procedures used		
RCTs		
Randomisation	Sequence Generation	
	Type	
	Allocation Concealment	
	Implementation	
Blinding	Participants	
	Data Collectors	
	Data Analysts	
Incomplete outcome data (attrition and exclusions)		
Selective reporting		
Other sources of bias		

Participant Characteristics	
Age	
Gender	
Ethnicity	
Depression scores	
Method of assessment/diagnosis of depression	
Chronic physical illness	
Other comorbidities	

Intervention Components	
BA Components	
Mode of delivery	

Delivered by	
Training received by practitioner delivering intervention	
Individual/group sessions	
Group size for group-based intervention	
Duration of intervention	
Number of sessions	
Length of sessions	
Treatment setting	
Manualised treatment	Yes No
Measurement of treatment integrity	Yes No
Adaptations to intervention	
Type of control condition	

Outcome Measurements	
Primary outcome measurements	
Quality of primary outcome measurements	
Length of follow-up	

Statistical Techniques	
Power calculation	
Method of dealing with missing data	
Length of follow-up	

Participant Flow	
Randomised to intervention	
Randomised to control	
Lost to follow-up intervention	
Lost to follow-up control	
Analysed intervention	
Analysed control	

Results	
**BA Intervention**	
Outcome measurement	
Pre-intervention means	
Pre-intervention standard deviation	
Pre-intervention number analysed	
Post-intervention means	
Post-intervention standard	

deviation	
Post-intervention number analysed	
**Control**	
Outcome measurement	
Pre-intervention means	
Pre-intervention standard deviation	
Pre-intervention number analysed	
Post-intervention means	
Post-intervention standard deviation	
Post-intervention number analysed	
**Qualitative Research**	
Main themes	
Main sub-themes	
**Observations**	
Observed results	

Additional Comments
